# Polarization-Dependent Plasmon-Induced Doping and Strain Effects in MoS_2_ Monolayers on Gold Nanostructures

**DOI:** 10.1021/acsnano.4c13867

**Published:** 2025-01-10

**Authors:** Matheus Fernandes Sousa Lemes, Ana Clara Sampaio Pimenta, Gaston Lozano Calderón, Marcelo A. Pereira-da-Silva, Alessandra Ames, Marcio Daldin Teodoro, Guilherme Migliato Marega, Riccardo Chiesa, Zhenyu Wang, Andras Kis, Euclydes Marega Junior

**Affiliations:** † Instituto de Física de São Carlos, Universidade de São Paulo, São Carlos 13566-590, Brazil; ‡ Departamento de Física, Universidade Federal de São Carlos, São Carlos 13565-905, Brazil; § Institute of Electrical and Microengineering, École Polytechnique Fédérale de Lausanne Lausanne 1015, Switzerland; ∥ Institute of Materials Science and Engineering, École Polytechnique Fédérale de Lausanne, Lausanne 1015, Switzerland

**Keywords:** 2D material, TMD, plasmonics, light–matter interaction, strain, doping

## Abstract

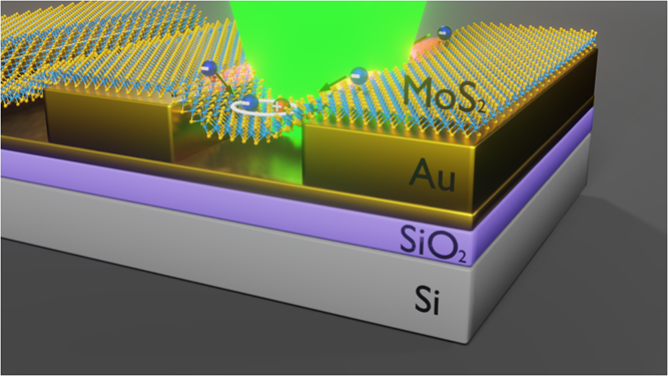

Monolayers of transition-metal dichalcogenides, such as MoS_2_, have attracted significant attention for their exceptional electronic and optical properties, positioning them as ideal candidates for advanced optoelectronic applications. Despite their strong excitonic effects, the atomic-scale thickness of these materials limits their light absorption efficiency, necessitating innovative strategies to enhance light–matter interactions. Plasmonic nanostructures offer a promising solution to overcome those challenges by amplifying the electromagnetic field and also introducing other mechanisms, such as hot electron injection. In this study, we investigate the vibrational and optical properties of MoS_2_ monolayer deposited on gold substrates and gratings, emphasizing the role of strain and plasmonic effects using conventional spectroscopic techniques. Our results reveal significant biaxial strain in the supported regions and a uniaxial strain gradient in the suspended ones, showing a strain-induced exciton and carrier funneling effect toward the center of the nanogaps. Moreover, we observed an additional polarization-dependent doping mechanism in the suspended regions. This effect was attributed to localized surface plasmons generated within the slits, as confirmed by numerical simulations, which may decay nonradiatively into hot electrons and be injected into the monolayer. Photoluminescence measurements further demonstrated a polarization-dependent trion-to-A exciton intensity ratio, supporting the hypothesis of additional plasmon-induced doping. These findings provide a comprehensive understanding of the strain-mediated funneling effects and plasmonic interactions in hybrid MoS_2_/Au nanostructures, offering valuable insights for developing high-efficiency photonic devices and quantum technologies, including polarization-sensitive detectors and excitonic circuits.

## Introduction

Two-dimensional (2D) materials have attracted significant attention from the scientific community since the discovery of graphene.^1−4^ Among these 2D materials, semiconducting transition-metal dichalcogenide (TMD) monolayers and their heterostructures are promising candidates for a variety of optoelectronic and nanophotonic applications.^5−10^ The optical properties of these semiconductors are dominated by excitonic effects because the reduced dielectric screening and quantum confinement at the monolayer limit allow for strong exciton binding energies even at room temperature.^11−14^ Although the strong excitonic effects in TMDs enable applications such as high-efficiency energy harvesting, light-emitting systems, and optical communications,^15−17^ their atomic thickness results in low light absorption and emission capacities. Additionally, the ability to manipulate exciton and trion transport is highly desirable for developing exciton-integrated circuits. Plasmonic nanostructures offer a way to improve both the emission of 2D-TMD by enhancing the electromagnetic field within their proximity^18,19^ and the exciton transport by strain-induced exciton drift.^20,21^

Considerable efforts have been devoted to study hybrid 2D-TMD/plasmonic devices. For example, Wang et al.^18^ demonstrated photoluminescence (PL) enhancement in the WSe_2_ monolayer suspended over sub-20 nm wide gold trenches, which support lateral gap plasmons. But due to the small trench width, no strain effects, such as drift-induced excitonic flux, were observed. Lee et al.^22^ reported the investigation of the exciton funneling effect for WSe_2_ and MoS_2_ in a gold nanogap geometry. Using tip-enhanced photoluminescence (TEPL), they showed enhanced exciton emission for WSe_2_ and exciton-to-trion conversion for MoS_2_ at the center of the nanogap. Nonetheless, they did not observe the formation of localized plasmons in the gold slits, which could result in hot electron injection from nonradiative plasmon decay. Koo et al.^23^ showed dynamic control of interlayer excitons and trions in a WSe_2_/Mo_0.5_W_0.5_Se_2_ heterobilayer using TEPL, achieving plasmonic hot electron injection and tunable trion conversion rates via an Au tip. While these studies highlight the potential of plasmon-enhanced TMD systems, tip-enhanced setups are inherently complex and challenging to implement,^24^ posing limitations for widespread adoption. Furthermore, from an industrial perspective, scalable and versatile devices with integrated plasmonic structures are essential for practical applications. These considerations underscore the need for research employing conventional spectroscopic techniques to elucidate the interactions between 2D-TMDs and integrated plasmonic systems.

In this work, we investigated a MoS_2_ monolayer (MoS_2_-ML) deposited on a gold substrate with a gold grating, examining the role of strain, doping, and plasmon formation on the vibrational and optical properties of the system. The strain-induced gradient in the slit, where significant deformation was observed, enables both exciton and charged carriers funneling to the nanogap center. By exploring the specific impacts of biaxial and uniaxial strain in the MoS_2_/grating hybrid system, we effectively captured the plasmonic effects of hot electron injection in a conventional micro-Raman spectroscopy setup. Numerical simulations and photoluminescence measurements were performed to further characterize the plasmonic behavior of the metallic grating, revealing that plasmons indeed play an important role in the observed optical properties. Our approach offers a simple and effective way to study the complex interactions in hybrid TMD/plasmonic systems, which could help to improve the development of novel optoelectronic applications, such as polarization-dependent photodetectors or carrier confinement devices.

## Results and Discussion

**Figure 1:**
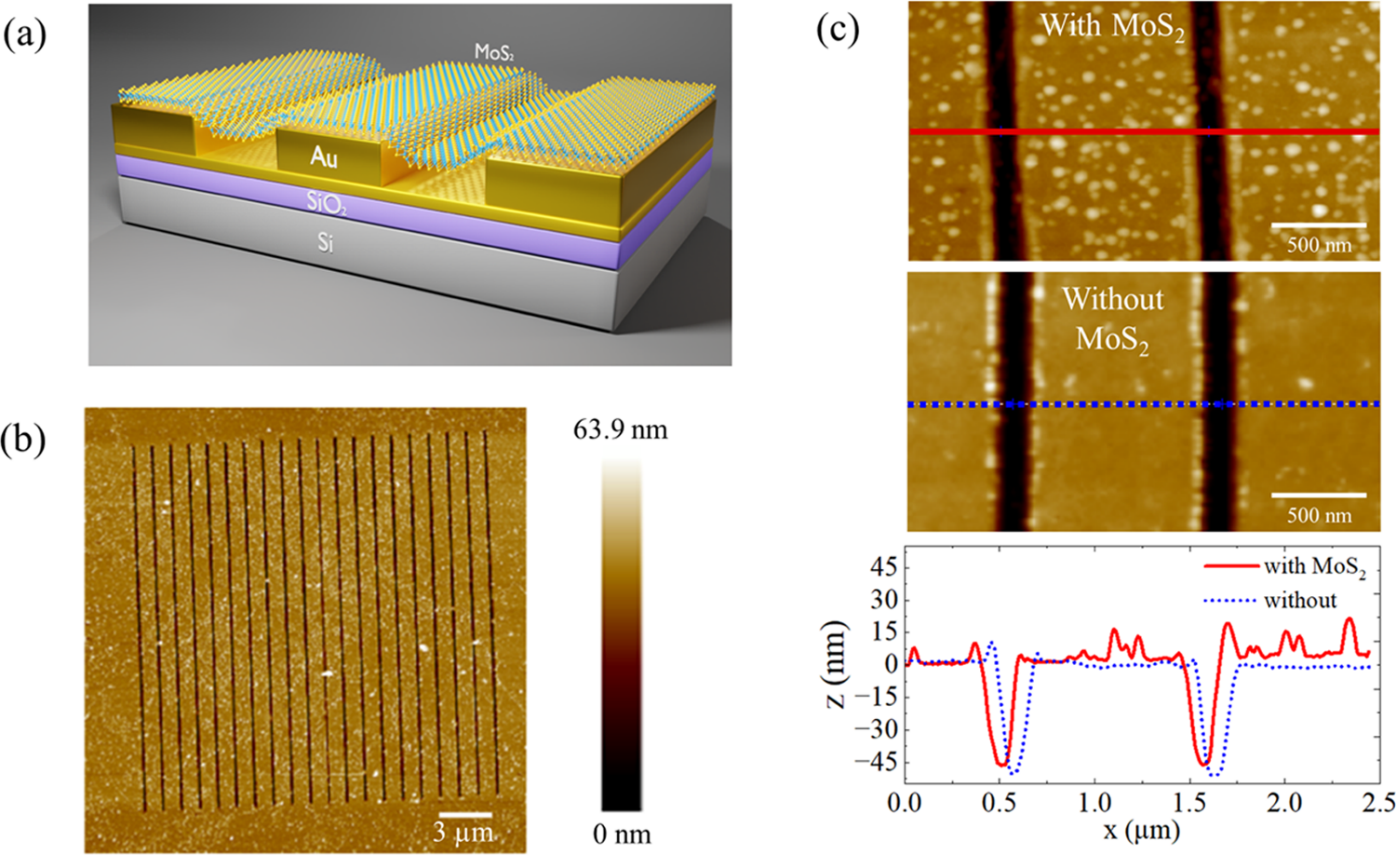
(a) Schematic representation of the sample. This diagram illustrates the MoS_2_-ML over the Au substrate and MoS_2_-ML over the Au grating regions as well as the suspended and supported ones. (b) Atomic force microscopy (AFM) image showing the topography of the entire structure. (c) Enlarged topographical images for the Au gratings with and without the MoS_2_ monolayer and their respective height profiles.

The MoS_2_ film was grown by metal–organic chemical vapor deposition (MOCVD) (for more details, see the Experimental Section). Although MoS_2_ monolayers obtained from mechanical exfoliation are generally of high quality, the process is challenging for industrial production. MOCVD-grown MoS_2_ films are more suitable for large-scale applications due to their scalability,^25,26^ with considerable efforts performed over the years to mitigate the inherent higher defect density originated from the growth process.^27,28^Figure 1(a) depicts a schematic out-of-scale illustration of the MoS_2_ monolayer/Au grating hybrid system where there are two regions of interest: MoS_2_-ML over the gold substrate and MoS_2_-ML over the gold grating. Sections of the monolayer in the grating region are in contact with the Au substrate (supported), whereas other parts are within the slit regions (suspended).

In Figure 1(b), one can see an AFM for the topography of the hybrid structure, where the periodic arrangement area of the Au grating is approximately 20 × 20 μm^2^. The geometric parameters of the gratings are 52 nm of slit depth, 120 nm of slit width, and 1075 nm of periodicity (for more details, see Section 1 in the Supporting Information). These parameters were chosen because of the following aspects. First, we were interested in this study in probing only one slit, resulting in the choice of an array with the highest grating periodicity: 1000 nm, which allowed the clear distinction of the slit and nonslit regions. Additionally, we wanted to simultaneously investigate the strain and plasmonic effects in such hybrid systems. Based on our numerical simulations of the electric field in the slits and recent works of the necessary strain to induce the exciton funneling effect,^22^ the choice of 100 nm in width and 50 nm in depth was made since it satisfied both conditions.

Figure 1(c) shows an enlarged AFM image of the Au grating with and without the MoS_2_ monolayer and their respective height profiles. It is possible to observe that the case of the Au grating with MoS_2_ possesses more roughness, which is associated with residues of monolayer transfer processes. Although the necessary procedures were performed to remove the unwanted residues (Experimental Section), we were not able to achieve complete mitigation. Nevertheless, one can see that the monolayer appears to match the shape of slits, even if a conclusive statement is not possible only with this measurement due to the tip–sample convolution effects.^29^

Analyzing the graph in Figure 1(c), we measured a large deformation in the grooves with a maximum value at the center of the monolayer of approximately 46 nm. This value exceeds what was reported for MoS_2_-ML over Si grating,^30^ and it surpasses that reported in other studies involving monolayers over Au nanostructures, which typically range within a few dozen nanometers.^22^ Although large vertical deformations may cause an excessive strain in the MoS_2_-ML and lead to phase transitions, our measurements support the idea that the 2D material is undamaged and is still in the 2H crystalline phase (see Section 2 in the Supporting Information). Moreover, it is important to note that during the transfer process, a competition between the surface adhesion energy and the bending energies of the monolayer occurred.^31^ Thus, the strong interaction between the sulfur atoms and gold atoms,^32−36^ along with the high elastic response of the MoS_2_ monolayer,^37^ culminated in the monolayer conforming to the periodic nanostructure.^31^

**Figure 2:**
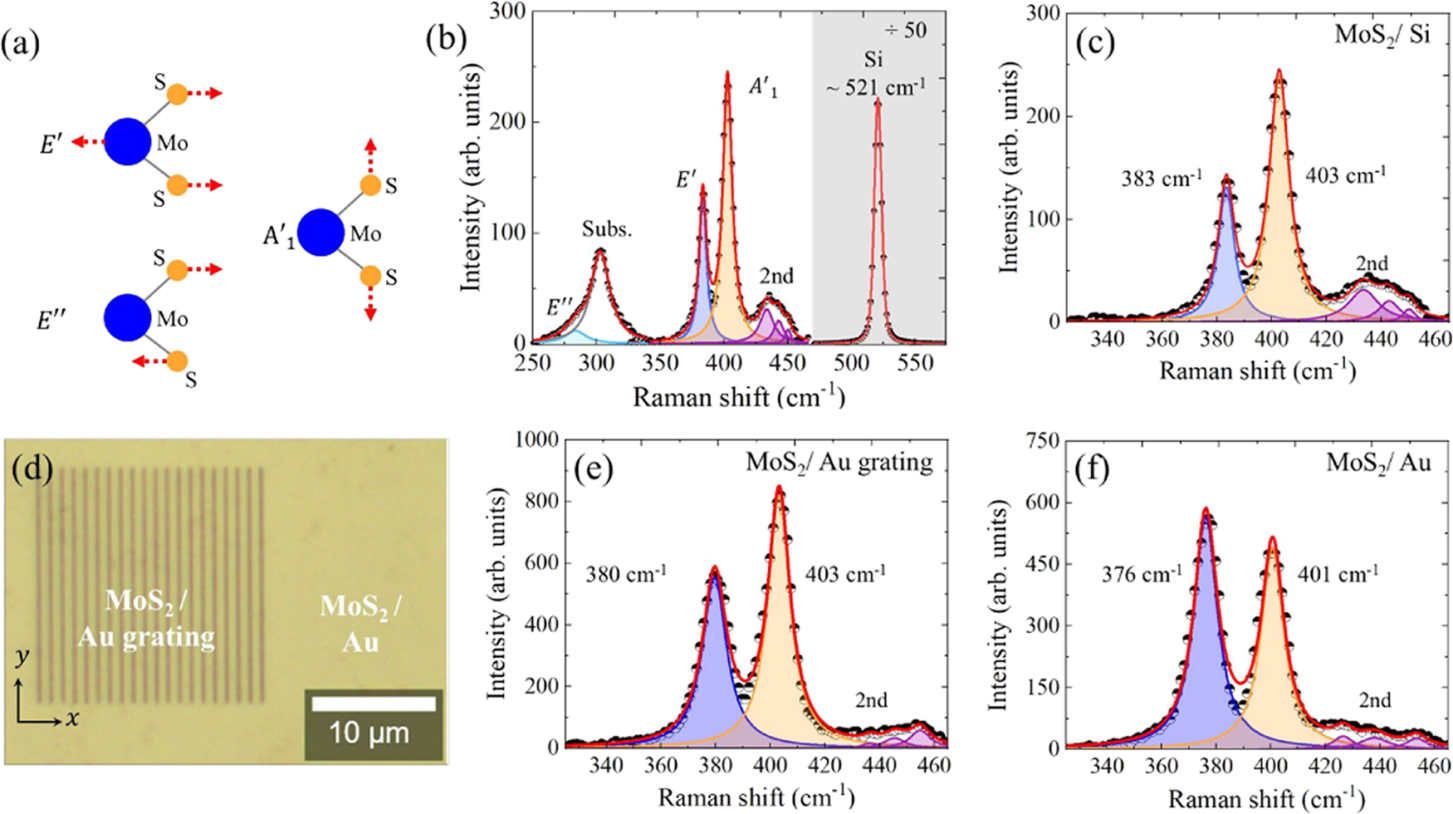
(a) Displacement schematics of the Raman-active vibrational modes for MoS_2_ monolayers (ML). (b) Raman spectrum of MoS_2_/Si, illustrating the sample modes and (c) a spectrum from a specific range of interest, showing the peak position of MoS_2_ on the Si substrate. (d) Optical image of the sample, highlighting the MoS_2_/Au grating and MoS_2_/Au regions, with their respective spectra in (e, f).

Once the morphology of the hybrid structure was studied, Raman spectroscopy was performed to investigate how the vibrational properties of the monolayer change in the different regions of the sample. The most important Raman-active modes for 2H-MoS_2_ monolayers (TMD monolayers in general) at the Γ point—which is the only permitted phonon wavevector for first-order Raman processes when using visible light excitation (λ = 532 nm)^38^—are the in-planes *E*′, *E*″, and out-of-plane *A*_1_^′^ modes,^39^Figure 2(a). Moreover, the *E*′ and *A*_1_^′^ peak positions show opposite trends as a function of the thickness, allowing the layer number identification through their relative frequency difference.^38,40^Figure 2(b–f) shows the Raman spectra of the MoS_2_-ML transferred over the SiO_2_/Si substrate (MoS_2_/Si) as well as the Au substrate (MoS_2_/Au) and Au grating (MoS_2_/Au grating). In addition, an optical image is presented in Figure 2(d), showing the measured regions in the sample, corresponding to MoS_2_/Au and MoS_2_/Au grating. It is important to note that due to the spot size limitation, the Raman spectrum in the Au grating region will reflect a combination of both suspended and supported regions. In every region, we observe a typical Raman spectrum composed of the in-plane and out-of-plane modes, including the second-order peaks (second), that have been reported for this excitation condition.^41^ The Raman spectrum in the MoS_2_/Si region in Figure 2(c) reveals a frequency difference of (ω_*A*_1_^′^_ – ω_*E*′_) ∼ 20 cm^–1^ between the *A*_1_^′^ and *E*′ modes, confirming that the MoS_2_ transferred to the substrate is indeed a monolayer.^38,40^ For the other regions, Figure 2(e,f), we can see that the peak positions of the *A*_1_^′^ and *E*′ modes change in comparison to the MoS_2_/Si. In particular, the *E*′ mode exhibits a significant red shift (the Raman shifts are lower), while *A*_1_^′^ shows a slight change. Consequently, each mode experiences a distinct shift, resulting in a greater frequency separation in both cases compared with the MoS_2_/Si, with the MoS_2_/Au region displaying a more pronounced effect.

**Figure 3:**
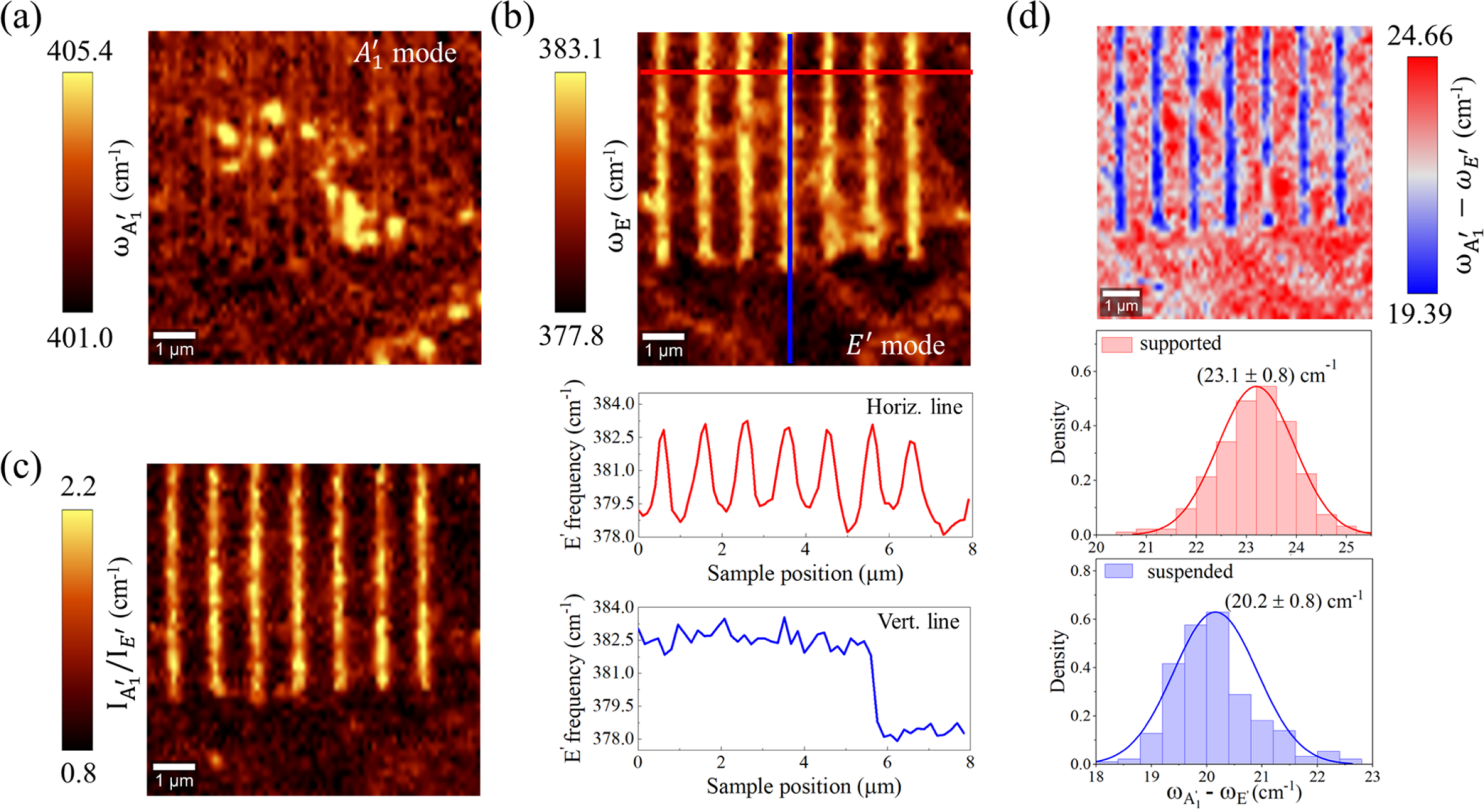
Color maps of the (a) *A*_1_^′^ and (b) **E**′ MoS_2_ vibrational modes. The red and blue lines are graphically represented below for the *E*′ mode. (c) Color map of the ratio between the intensities of *A*_1_^′^ and *E*′ modes. (d) Color map related to ω_*A*_1_^′^_ – ω_*E*′_ and the normal distributions for the supported and suspended regions in the sample.

In order to extract relevant statistical information on the spatial distribution of the frequency shifts, intensities, and ω_*A*_1_^′^_ – ω_*E*′_, including separate analyses of the supported and suspended regions on the grating, we performed Raman hyperspectral maps. In Figure 3, one can see the color maps of the (a) *A*_1_^′^ and (b) *E*′ peak frequencies as a function of the position. It is clear that the in-plane mode is well modulated by the grating pattern, as shown by the graphical analysis along the two lines indicated in Figure 3(b). In both supported (in regions in contact with Au inside of the grating and outside it) and suspended regions, the *E*′ peaks are red-shifted in comparison to the MoS_2_/Si, and these shifts are larger for the supported case, as previously observed for the single Raman spectra in Figure 2. In contrast, the peak position of the out-of-plane lattice vibration shows a less pronounced correlation with the supported and suspended regions as compared with the *E*′ mode, where a clear distinction between these two regions cannot be made. However, the *A*_1_^′^ peaks also show a slight red shift relative to the silicon case. Considering these results extracted from hyperspectral analysis, we noted that the red shift behavior of the peak frequencies corresponds to a global feature of the sample, resulting in a higher separation between out-of-plane and in-plane modes as previously reported in the literature.^33^ More specifically, we obtained Raman hyperspectral measurements (ω_*A*_1_^′^_ – ω_*E*′_) = (23.1 ± 0.8) and (20.2 ± 0.8) cm^–1^ for supported and suspended regions, respectively, as shown in Figure 3(d).

Furthermore, as we can observe in Figure 3(c), the ratio between the mode’s intensities (*I*_*A*_1_^′^_/*I*_*E*′_) varies more than twice comparing the suspended and support regions. Among all of the effects, the most prominent is related to the interaction between the substrate and the monolayer. Although the Au substrate affects the mode displacement of both modes, we have found that the amplitude of the *A*_1_^′^ mode is more sensitive to this effect (more details in Section 4 in the Supporting Information). This behavior can be explained by the strong interaction between the Au and S atoms,^32−36^ which restricts the amplitude of the out-of-plane lattice vibration when the MoS_2_ is in contact with the Au substrate. For the suspended regions, the Au–S interaction decreases and allows the proper mode displacement for the *A*_1_^′^ mode, resulting in a greater intensity.

Considering all of the characteristics present in the studied sample—such as the lattice mismatch between the MoS_2_ monolayer and the substrate,^42^ significant monolayer deformation within slits, intrinsic doping of the monolayer, potential charge exchange between the monolayer and substrate,^43^ and the plasmonic nature of the metallic grating—it is suggested that substantial strain and doping effects may arise. Consequently, these effects could be responsible for altering the amplitude and frequency of the Raman modes.^34,43−46^ First, focusing on strain, we assume that the monolayer undergoes elastic deformation without any permanent changes to its lattice, which is a reasonable assumption given the aforementioned factors. The general equation to quantify the change in frequency of a particular mode Δ*ω*_m_ = ω_m_ – ω_m_^0^ as a function of the strain, where ω_m_^0^ is the reference frequency, is given in terms of Grüneisen parameter γ_m_ and the shear deformation potential β_m_ as follows^44,47^
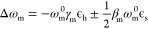
1where ϵ_h_ = ϵ_ll_ + ϵ_tt_ is the hydrostatic component and ϵ_s_ = ϵ_ll_ – ϵ_tt_ is the shear component of the applied strain, with ϵ_ll_ the longitudinal and ϵ_tt_ the transversal components.

However, it should be pointed out that we need to distinguish between the types of mechanical deformation present in the supported and suspended regions of the sample. In the supported regions, inside and outside the grating, the strain is biaxial, meaning it is imposed in both directions within the monolayer plane. This strain is a consequence of the different lattice parameters between MoS_2_-ML and Au film, affecting the arrangement of the monolayer over the gold substrate^42^ and deforming the monolayer in both armchair and zigzag directions. In this case, we have ϵ_ll_ = ϵ_tt_ = ϵ, so eq 1 for a biaxial strain reduces to^44^
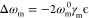
2On the other hand, in the suspended regions within the grating, the monolayer undergoes uniaxial strain as a result of the competition between adhesion and bending, leading to its conformation within the slits.^48^ We expect a more complex strain distribution along the monolayer plane with different local strain magnitudes in which the center experiences maximum compressive strain and the areas near the corners of the slits undergo maximum stretching. Nevertheless, despite the MoS_2_-ML experiencing inhomogeneous strain, the limitation of the laser spot only permits the analysis of the average strain in the suspended regions. Thus, we are able to probe only the average uniaxial strain. In this situation, we have ϵ_ll_ = ϵ and ϵ_tt_ = −*ν*ϵ, where ν is the Poisson’s ratio.^44^ Then, eq 1 for uniaxial strain is given by
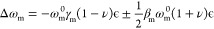
3In addition to the strain, the doping effect can also alter the peak positions of the Raman modes. This effect is accounted for by a factor of *k*_m_*n*, where *k*_m_ is the shift rate of Raman peak as a function of the electron concentration *n*.^43,49^ Including that in eqs 2 and 3, we have that the final expression for the changes in the Raman frequency due to a biaxial strain and doping is expressed by
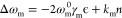
4whereas in the case of an uniaxial strain, it is given by
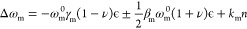
5

An effective method for quantitatively evaluating the contributions of strain and doping mechanisms on the frequency changes of the Raman modes is to construct a correlative plot of the *A*_1_^′^ versus *E*′ peak frequencies. Such an approach has been successfully applied to the characterization of the 2D and G peaks in graphene^44,45,50^ and has been recently used for MoS_2_ monolayer as well.^49,51,52^ Thus, from the respective color maps for each Raman mode, exposed in Figure 3, we extracted the coordinate (*E*′, *A*_1_^′^) and obtained the experimental graphical correlation between the out-of-plane and in-plane frequency modes in the supported and suspended regions. For comparison, the relation *A*_1_^′^ vs *E*′ peak frequencies for the MoS_2_ monolayer deposited over Si was also obtained. Finally, we carried out Raman scattering measurements, exciting the sample with incident radiation polarized parallel (*E*_∥_) or perpendicular (*E*_⊥_) to the slits.

To help the understanding of this plot, parallel lines corresponding to constant biaxial or uniaxial strain and constant doping were drawn, which can be calculated by fixing the values of ϵ or *n* in eqs 4 and 5 and solving the linear set of equations for the *A*_1_^′^ and *E*′ modes. These theoretical values were calculated using reported values for each parameter. For the biaxial strain in the supported regions, we used γ_*E*^′^_ = 0.68 and γ_*A*_1_^′^_ = 0.21 for the Grüneisen parameters for the *E*′ and *A*_1_^′^ modes of MoS_2_ monolayer, respectively.^53^ On the other hand, for the uniaxial strain in the suspended regions, to the best of our knowledge, only the Grüneisen parameter γ_*E*^′^_ = 1.1^54^ and shear deformation potential β_*E*^′^_ = 0.78^54^ for the *E*′ mode is known, since the *A*_1_^′^ mode is essentially insensitive to the applied strain.^55^ The MoS_2_-ML Poisson’s ratio used was ν = 0.29, as reported by Cooper and his collaborators.^56^ In addition, to calculate the electron doping terms, the shift rates employed were *k*_*E*′_ = −0.33 × 10^–13^ cm and *k*_*A*_1_^′^_ = −2.2 × 10^–13^ cm for in-plane and out-of-plane modes, respectively.^43^ Finally, the reference frequencies for the peak positions ω_m_^0^ of the *E*′ and *A*_1_^′^ Raman modes are obtained from an unstrained, suspended MoS_2_ membrane, where the substrate effects can be excluded and the deformations can be neglected. The values are ω_*E*′_^0^ = 385 cm^–1^ and ω_*A*_1_^′^_^0^ = 405 cm^–1^ as documented by Lloyd et al.^53^

**Figure 4:**
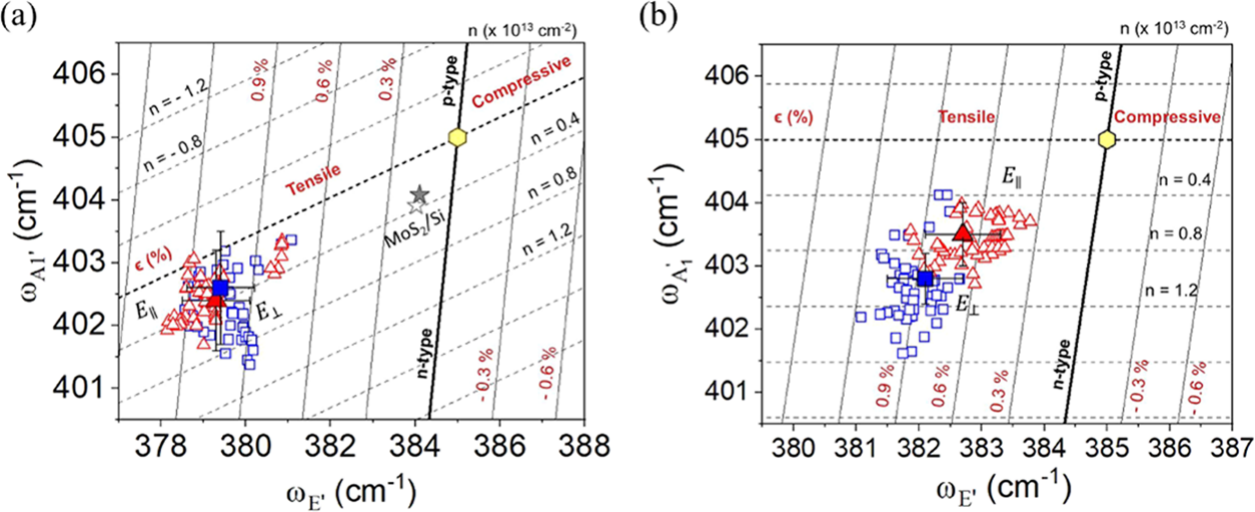
Correlative plot of the *A*_1_^′^ versus *E*′ for (a) biaxial and (b) uniaxial strains applied to the sample. The plots show the experimental points and their averages for the supported and suspended regions. The yellow hexagon corresponds to the reference MoS_2_-ML, the red triangles for the configuration *E*_∥_, and the blue squares the *E*_⊥_.

Figure 4(a,b) shows the correlative graph of the peak frequencies of *A*_1_^′^ versus *E*′. It is possible to see the experimental data for each region and polarization represented as scatter plots in the ϵ – *n* plane. The straight bold line depicts the case for ϵ = 0, and the other black lines parallel to it relate to ±0.3% variations in strain calculated as previously discussed. Correspondingly, the dashed bold line represents the case for *n* = 0, and the other dashed lines are associated with ±0.4 × 10^13^ cm^–2^ variations in the electron doping. The lines of zero strain and zero doping cross at the point (385, 405) cm^–1^ depicted as a yellow hexagon in both graphs, which represents the aforementioned reference case of free-standing MoS_2_-ML. Figure 4(a) corresponds to the region of biaxial strain, where the hollow blue squares represent experimental points to condition*E*_⊥_ and the hollow red triangles represent *E*_∥_. Additionally, as MoS_2_-ML over Si undergoes biaxial strain, it is represented in the same graph by hollow and filled stars corresponding to *E*_⊥_ and *E*_∥_, respectively. Similarly, Figure 4(b) shows the *A*_1_^′^ versus *E*′ peak frequencies for uniaxial strain in the suspended regions, following the same pattern of Figure 4(a). Also, we observe that the filled square and triangle symbols in both graphs in Figure 4(a,b) correspond to the mean value of the normal distributions associated with those experimental points, and the error bars represent their standard deviation.

From Figure 4(a), the MoS_2_ monolayer deposited over SiO_2_/Si experiences, as expected, a slight mean tensile strain. The average value is ϵ = 0.2%, which might be related to the surface adhesion properties of MoS_2_ monolayer over the Si substrate,^31^ but is lower than observed in the literature.^49^ In addition, we noticed an average electron density around *n* = 0.4 × 10^13^ cm^–2^ corresponding to an n-type doped film. This can be explained by the natural propensity of MoS_2_ monolayer to exhibit n-type conductivity when grown via MOCVD methods, where point defects present during the growth process provide an effective donor level near the conduction band minima.^57^ Additionally, as reported by Chae et al.,^49^ the silicon substrate can significantly dope the MoS_2_ with n-type. Concerning the polarization, we do not observe any significant dependence, as both points are positioned almost identically (see Section 3 in the Supporting Information for more details).

For the supported regions corresponding to MoS_2_-ML in contact with the Au substrate (both the inside and outside areas of the grating), it can be seen that the data cloud is located in a region of the ϵ – *n* plane equivalent to a tensile biaxial strain and n-type doping. The average tensile strains and electron concentrations for the supported regions are ϵ = 1.0% and around *n* = 0.4 × 10^13^ cm^–2^ for both incident fields *E*_⊥_ and *E*_∥_. A tensile biaxial strain at the interface originates from the lattice mismatch between the monolayer and Au, in which the lattice parameter of Au is about 5.4% larger than MoS_2_-ML.^42^ Other authors have measured strains similar to ours for the MoS_2_-ML/Au system, where large ϵ values up to 1.2% have been reported.^34^ Interestingly, we noted n-type doping for the supported region, which is comparable to MoS_2_/Si, in contrast to previous works that observed an electron transfer from monolayer to Au.^58^ However, it should be noted that the substrate roughness plays an important role in determining the contact behavior between MoS_2_-ML and the underlying material. In our case, the measured root-mean-square (RMS) roughness for the Au substrate in the vicinity of the grating was 1.45 nm (see Section 1 in the Supporting Information). This roughness could prevent proper contact between the monolayer and the metal, reducing the efficiency of charge transfer, as has been observed in other studies that reported n-type doping in MoS_2_/Au systems.^34^ Consequently, our results suggest that the natural n-type doping of MoS_2_-ML is preserved.^59^ Finally, the MoS_2_/Si and MoS_2_/Au regions are not close to each other, so variations in hydrogen gas concentrations could occur, which would also influence doping levels along the sample.

The plot of the peak frequencies *A*_1_^′^ versus *E*′ for suspended regions is shown in Figure 4(b), where the MoS_2_ monolayer is now subject to a uniaxial strain. From the graph, we note that the experimental data are located in a region of the ϵ – *n* plane corresponding to a tensile uniaxial strain and n-type doping for both polarizations. However, differently from what was observed for the supported regions and MoS_2_/Si, we identified a polarization dependence in the strain and doping values. The magnitude of uniaxial deformation in the suspended regions varies by approximately 0.2% between each polarization condition, with a higher magnitude for the *E*_⊥_ (ϵ = 0.9%) than for the *E*_∥_ (ϵ = 0.7%) condition, which corresponds to the application of the strain direction.

Previous works have demonstrated that the in-plane uniaxial strain applied parallel to the MoS_2_ bonds can lift the degeneracy of the *E*′, splitting it in *E*′^–^ and *E*′^+^ modes, and these modes possess a polarization dependence.^60,61^ In our measurements, we did not observe a noticeable splitting of the *E*′ mode, only noticing an increase in the line width of these peaks in the slit regions for both polarization conditions (see Section 4 in the Supporting Information). As it is not possible to determine the direction of the strain relative to the crystallographic directions, we can only suggest from our results that the uniaxial strain induces anisotropy in the MoS_2_-ML lattice, as reported in the literature.^61^ Concerning the electron concentration in the sample, one can see that the *n* value in the slits is higher than those obtained for the supported regions, which is consistent with the fact that the MoS_2_ monolayer has less contact with the gold surface, decreasing any charge transfer mechanism. Moreover, the uniaxial strain-induced gradient in the nanogap is responsible for the funneling effect, where the excitons and electrons drift to the center of suspended regions.^22^ Finally, we observed a polarization-dependent doping effect in the suspended regions, with a higher doping level for *E*_⊥_ (*n* = 1.0 × 10^13^ cm^–2^) than the *E*_∥_ (*n* = 0.7 × 10^13^ cm^–2^) case, indicating that in this configuration, an additional source of charge is induced.

**Figure 5:**
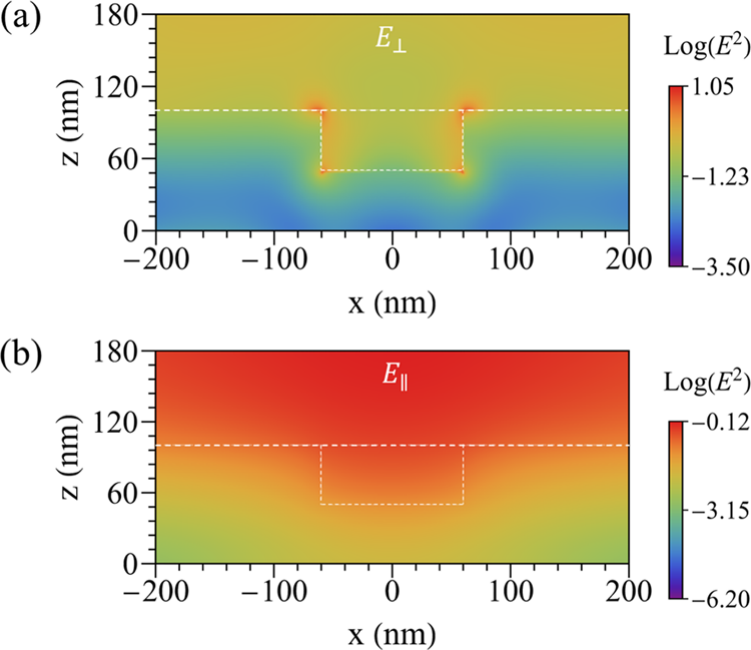
Electromagnetic field distribution and its corresponding graphical representation for incident radiation at 532 nm polarized perpendicular (a) and parallel (b) to the slits, respectively.

The Au grating supports plasmon modes that are closely correlated with the polarization direction of the incident field relative to the orientation of the slits.^62^ To substantiate this hypothesis within our experimental framework, numerical simulations were performed, revealing distinct behaviors in the electric field distribution when the incident radiation is linearly polarized perpendicular (*E*_⊥_) or parallel (*E*_∥_) to the slits, as depicted in Figure 5. One can see that localized surface plasmons (LSPs) originate when the polarization of the incident radiation is perpendicular to the grating, where the plasmon field distribution concentrates at the corners of the slits (we have considered regular corners; for more details, check Section 5 in the Supporting Information), and can interact with the MoS_2_-ML, as seen in Figure 5(a). In contrast, when the incident radiation is parallel to the grating, no LSP modes are observed. Instead, the nanostructure reflects much of the radiation, as illustrated in Figure 5(b). Furthermore, the maximum value of the squared electric field at the Au-MoS_2_ interface for *E*_⊥_ is approximately 25.3 times larger than that for *E*_∥_. Following the generation of LSPs, these plasmons decay through various mechanisms, such as phonon, defect, electron–electron scattering-assisted absorption, and Landau damping, producing “hot carriers”.^63^ These hot carriers (in our case electrons) may possess sufficient energy to overcome the Schottky barrier between the Au grating and MoS_2_-ML, facilitating effective charge injection into the adjacent monolayer, as reported in refs (64−66). Once injected into the semiconductor, these hot electrons undergo the same funneling effect previously discussed and drift to the nanogap center. Thus, the injection of hot electrons into MoS_2_-ML may explain the polarization-dependent doping levels observed in the suspended regions under our experimental conditions.

**Figure 6:**
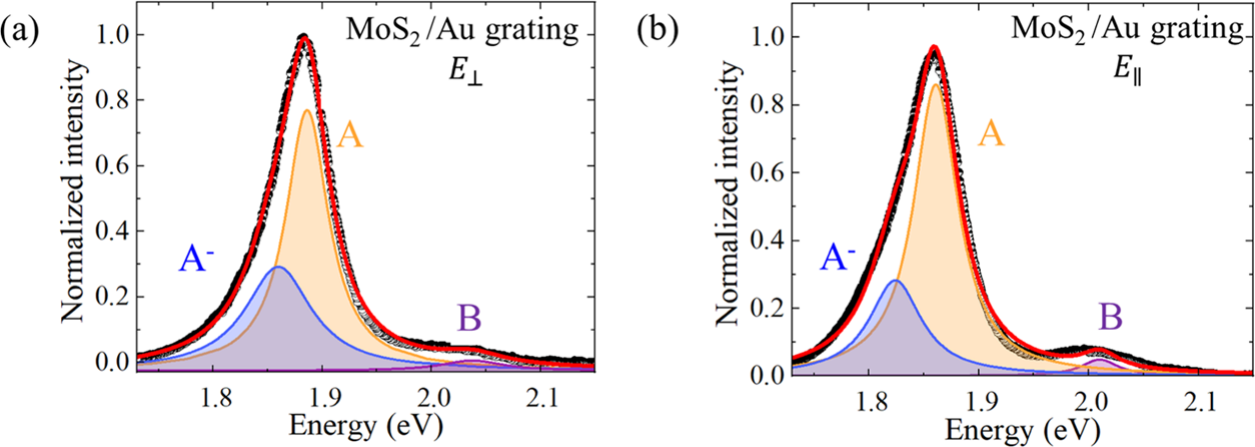
Normalized PL for MoS_2_/Au grating for radiation polarized (a) perpendicularly and (b) parallel to slits, respectively.

To elucidate this idea, photoluminescence measurements were performed to understand the effect of plasmon perturbations on the optical properties of the exciton and trion states of the MoS_2_ monolayer. Figure 6 shows the normalized PL spectra for MoS_2_-ML deposited on the metallic grating for each incident polarization condition. Notably, three peaks can be identified, corresponding to the A and B neutral excitons resulting from direct transitions between the valence and conduction bands at the K point, and the negative trion (A^–^ ↔ A^0^ + e^–^), the formation of which is influenced by the availability of free electrons in the system.^67^

From the fitting of the emissions in each sample region, we computed the ratio between the trion intensity and the overall emission related to the A exciton, which represents the most significant emission. The formula is given by η = *I*_*A*^–^_/(*I*_*A*^–^_ + *I*_*A*_),^68^ and for both cases, we observe a significant η. The high population of trions in MoS_2_ can generally be explained by the natural doping of the monolayer due to the growth process, as mentioned earlier. Furthermore, it is associated with the funneling effect that enhances the conversion of A excitons to trions in nanogap regions.^22^ But most importantly, the intensity ratio between the trion and the A exciton is slightly greater when the grating is perpendicular (η = 38.2%) to the incident radiation than when it is parallel (η = 31.9%). This indicates a higher density of free electrons for the first configuration, which facilitates the generation of charged excitons. Therefore, this result supports the hypothesis of a plasmon-induced hot electron injection mechanism.

## Conclusions

In this study, we explored the physical and optical properties of MoS_2_ monolayers grown by MOCVD and deposited on silicon and gold substrates as well as nanostructured gold gratings. Our analysis revealed that the monolayer conforms sharply to the grating slits, a behavior governed by the interplay between adhesion and bending energies during the transfer process. Raman spectroscopy enabled us to disentangle the contributions of strain and doping to the vibrational mode frequency shifts, considering the distinct effects of biaxial and uniaxial strain in the supported and suspended regions, respectively. By correlating the *A*_1_^′^ and *E*′ mode frequencies, we estimated the strain and carrier concentration, uncovering a significant polarization-dependent electron concentration in the MoS_2_ monolayer. This polarization dependency is attributed to hot electron injection into MoS_2_-ML, facilitated by nonradiative decay of localized plasmons in the gold gratings. Numerical simulations confirmed the generation of localized plasmons under our experimental conditions, further supporting the role of plasmonic effects. Photoluminescence measurements validated these findings, showing an enhanced trion population consistent with plasmon-induced doping. These results provide valuable insights into the interplay among strain, doping, and plasmonic interactions in hybrid MoS_2_/Au systems. By leveraging strain-mediated exciton and electrons funneling along with plasmon-induced carrier dynamics, our work contributes to advancing the understanding of TMD monolayers integrated with plasmonic nanostructures. This knowledge paves the way for designing novel optoelectronic and quantum devices.

## Experimental Section

### Sample Preparation

#### Nanostructure Fabrication

The electron beam (e-beam) evaporation technique deposited a 100-nm-thick gold film over the SiO_2_/Si substrate. After the thermal evaporation step, e-beam lithography combined with ion-beam-assisted etching was used to fabricate the metallic square gratings. First, the poly(methyl methacrylate) (PMMA) resist is spin-coated onto the Au film. Then, the resist-coated sample is loaded into an electron beam lithography system, where the exposed regions of the resist undergo a chemical change, making them more soluble. After exposure, an appropriate developer solution removes the soluble regions, creating a patterned resist layer. The patterned resist serves as a mask for ion-beam etching (Nexus IBE350 from Veeco), allowing for removal of the gold material and formation of the desired structure. Finally, the leftover PMMA from the sample is removed using acetone, isopropyl alcohol, and deionized water, resulting in the final patterned structure.

#### MoS_2_ Monolayer

Large-area MoS_2_ film was grown by MOCVD. Before growth, c-plane sapphire substrate wafers are annealed at approximately 1000 °C in the air and treated with potassium hydroxide (KOH). Then, an aqueous solution of sodium molybdate (Na_2_MoO_4_) is spin-coated onto the substrate, which provides the molybdenum (Mo) precursor and alkali metal promoter (Na) for monolayer growth and placed in the growth chamber. Finally, the sulfur (S) precursor is delivered to the quartz tube by liquid diethyl sulfide ((C_2_H_5_)_2_S) with argon and hydrogen as the carrier gases. The reaction mechanism occurs at a growth temperature of 850 °C for about 20 min, allowing the formation of a large-area continuous film.

#### Transfer Procedure

The MOCVD-grown material is transferred to the gold gratings by delamination using thermal release tape (TRT) and PMMA as a support layer. Initially, the PMMA is spin-coated onto the wafer containing the MoS_2_ monolayer and heated at 180 °C. Next, a TRT with the same size as the grown material is attached to the MoS_2_ sample. It is important to note that using a TRT provides a strategy to delaminate TMD layers without using etchants or solvents that render the substrate unusable for another growth process. Then, the PMMA/MoS_2_/sapphire sample is placed in deionized water, which can penetrate between the TMD layer and the sapphire substrate by scratching the corners of the film. The PMMA/MoS_2_ system is lifted off, transferred manually to the target substrate, and put in a hot plate at 135 °C for removal of the thermal release tape. Finally, the surface was cleaned by acetone and isopropanol and then dried using nitrogen gas.

### AFM

AFM measurements were performed using Bruker’s Dimension Icon AFM in intermittent contact mode, with a nominal cantilever spring constant of 26 N/m and a nominal resonance frequency of 300 kHz. The silicon tip had an approximate radius of 7 nm.

### Raman Analysis

The Raman measurements were performed using a WITec alpha300 R confocal Raman microscope in the backscattering configuration and at room temperature. For the excitation source, a continuous-wave diode laser centered at 532 nm was used, which was focused onto the sample by a 100× objective lens with an NA = 0.9. The spot size of the laser is approximately 700 nm, and the laser power used was 3.92 mW. The spatial resolution of the system is ≈350 nm. The spectrometer comprises a 1200 grooves/mm diffraction grating (blaze at 500 nm) equipped with a charge-coupled device (CCD) photon detector. The Raman peaks were fitted using the Lorentzian function, according to expected Raman modes for MoS_2_. Moreover, the hyperspectral maps were acquired by using an electronically controlled piezoelectric stage. The Raman signals were calibrated using the silicon peak position (for our experiments, it was around 522 cm^–1^). All hyperspectral analyses were made using WITec Project FIVE 5.2 software. Finally, for the polarization-resolved measurements, the sample was rotated by using a manual rotation stage.

### PL Analysis

The photoluminescence spectra were recorded using a conventional PL setup. The excitation source was a laser centered at 532 nm, transmitted to the optical setup via a single-mode optical fiber. An aspheric lens with a 5 mm diameter, a focal distance of 1.6 mm, and a numerical aperture (NA) of 0.64 is used to focus the laser onto the sample and to collect the emitted signal (the spatial resolution is 700 nm). The sample is placed in a piezoelectric stage that controls the *x*, *y*, and *z* direction with a 10 nm spatial resolution. A multimode optical fiber sends the PL signal to a 150 lines/mm diffraction grating and then to a CCD photon detector. The spectra are acquired with an integration time of 10 s and 3 accumulations. All measurements were made at ambient conditions with a laser power of approximately 3.9 mW. Besides, to obtain the PL hyperspectral microscopy image, a ZEISS LSM780 confocal microscope was used with an objective lens 20X/NA = 0.8, a diode laser centered at 405 nm, and laser power around 13 μW.

### Numerical Simulations

Numerical simulations of the electromagnetic fields were implemented using the commercial software Ansys Lumerical finite-difference time-domain (FDTD). Symmetric boundary conditions were applied in the *x* and *y* boundaries to reduce computational cost. Perfectly matched layers (PMLs) were used on the *z* boundary to eliminate unwanted reflections at the interface. Furthermore, a Gaussian beam, centered at 532 nm, with a high NA was used as the excitation source to closely mimic the experimental conditions in the simulation. The MoS_2_ monolayer thickness was fixed at 0.6 nm, and the wavelength-dependent permittivity data were obtained from ref (69). The mesh size d*z* = 0.02 nm was employed for the MoS_2_ region and the *z*-axis span of the simulation region between the nanostructure and the PML *z* boundary was larger than half of the source wavelength.

## Supporting Information Available

Additional experimental details; including the morphological features of the Au grating; the characterization of the MoS_2_ 2H-phase; the polarization dependence of MoS_2_ deposited over SiO_2_/Si; the intensity and line width maps from hyperspectral analysis; and the numerical simulations regarding grating protrusions (PDF)
