# Supplementary material for: Polarization-Dependent Plasmon-Induced Doping and Strain Effects in MoS2 Monolayers on Gold Nanostructures
Source: ACS Nano. 2025 Jan 10;19(2):2518–28. doi: 10.1021/acsnano.4c13867 (PMC11760179; doi:10.1021/acsnano.4c13867)
Supplement: Supplementary file 1 — nn4c13867_si_001.pdf [file nn4c13867_si_001.pdf]

Supporting Information: Polarization-Dependent Plasmon-Induced Doping and Strain Effects in MoS_2 Monolayers on Gold Nanostructures

Matheus Fernandes Sousa Lemes,[*,†,‖] Ana Clara Sampaio Pimenta,[†,‖] Gaston Lozano Calderón,[†] Marcelo A. Pereira-da-Silva,[†] Alessandra Ames,[‡] Marcio Daldin Teodoro,[‡] Guilherme Migliato Marega,[¶,§] Riccardo Chiesa,[¶,§] Zhenyu Wang,[¶,§] Andras Kis,[¶,§] and Euclydes Marega Junior[*,†]

†*Instituto de Física de São Carlos, Universidade de São Paulo, São Carlos, 13566-590, Brazil*

‡*Departamento de Física, Universidade Federal de São Carlos, São Carlos, 13565-905, Brazil*

¶*Institute of Electrical and Microengineering, École Polytechnique Fédérale de Lausanne Lausanne, 1015, Switzerland*

§*Institute of Materials Science and Engineering, École Polytechnique Fédérale de Lausanne, Lausanne, 1015, Switzerland*

‖*Authors are equally contributed*

E-mail: matheus.lemes@alumni.usp.br; euclydes@ifsc.usp.br

1. Morphological features of the Au grating

Figure 1 shows AFM analyses performed on the Au grating without a MoS_2 monolayer to obtain the geometrical parameters of the grating. We observe in figure 1 (a) that the Au grating alone exhibits less roughness compared to that measured for the Au grating with the MoS_2 monolayer. In fact, the root-mean-square (RMS) roughness for the Au substrate in the vicinity of the grating, figure 1 (d), was 1.45 nm, which is consistent with values reported in similar studies that observed n-type doping in MoS_2/Au systems.[1] This observation suggests that most of the roughness present in the hybrid system may originate from the transfer procedure, which leaves some residuals of PMMA and TRT. Figure 1 (b) presents an enlarged AFM image of a single slit where a redeposition of gold material is noticeable on the corners, which are unavoidable and are regularly observed in the literature for this type of nanoarray.[2] To measure the slit width without the presence of tip-sample convolution effects, we performed scanning electron microscopy (SEM) collecting the backscattered electrons, which yielded a width of 120 nm, as depicted in figure 1 (c). Regarding other morphological features, figure 1 (d) shows the AFM of a zoomed area in which the depth of the slit (d) and periodicity (p) were measured at approximately d = 52 nm and p = 1075 nm, as shown in figures 1 (e) and (f), respectively.

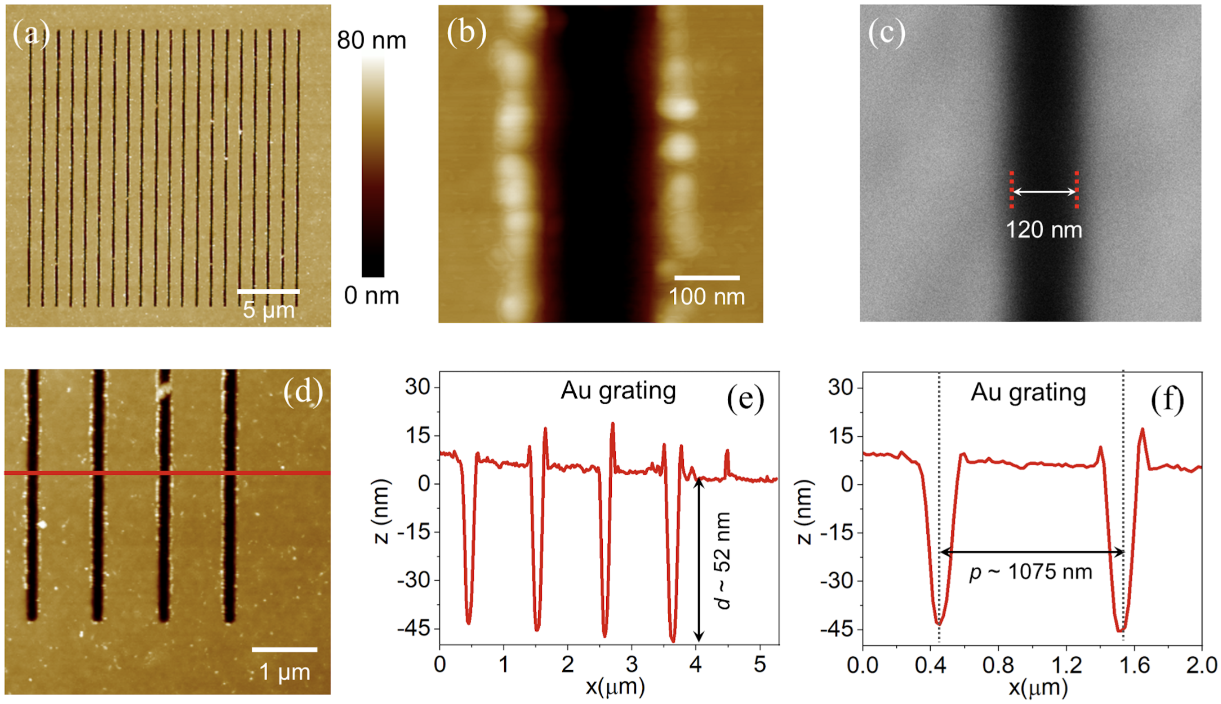

Figure 1: (a) Atomic force microscopy image analyzing the topographical profile of the sample without the MoS_2 monolayer. (b) An enlarged AFM image showing the gold protrusions on the corners of the slits. (c) Scanning electron microscopy (SEM) image, revealing the slit width. (d) Atomic force microscopy image presenting the profile of the slit arrays, analyzing the (e) depth and (f) periodicity of the slits.

2. Characterization of the MoS_2 2H-phase

In the literature, it was demonstrated that the MoS_2-ML achieved mechanical failures at vertical deflections of approximately 42 nm from nanoindentation experiments applying a loading force of around 180 nN.[3] Moreover, it is known that when the MoS_2-ML experiences excessive tension or compression, significantly distorting its lattice, a phase transition from the semiconductor phase 2H to the metallic phase 1T can occur.[4–6] All these effects mentioned change drastically the MoS_2-ML energy and phonon dispersion.[7] However, our characterizations in the grating region show the intense optical emission and Raman spectrum consistent with the semiconductor phase of the ML,[8] as depicted in figures 2 (a)-(c). It supports that the MoS_2-ML is undamaged and is still in the 2H crystalline phase, which suggests that the monolayer is in the elastic regime and with a slight suspension.

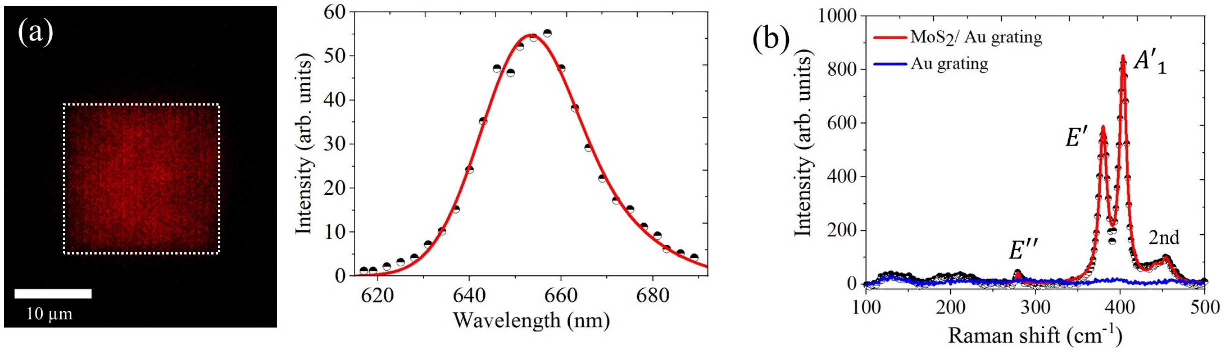

Figure 2: (a) Photoluminescence image of the MoS_2 on the Au grating, in which the region is delimited by the white square, and (b) its respective spectrum. (c) Ramam spectrum of the MoS_2 on the grating, exhibiting the most important vibrational modes to the 2H crystalline phase.

3. Polarization dependence of MoS_2 deposited over SiO_2/Si

Figure 3 shows, respectively, the Raman frequencies $\omega_{E'}$ and $\omega_{A_1'}$ (a), their full width at half maximum (FWHM) (b) and the difference $\omega_{A_1'}$ - $\omega_{E'}$ (c) as a function of the incident polarization angle for the MoS_2-ML deposited over SiO_2/Si. As can be seen, no polarization dependence is observed in this case.

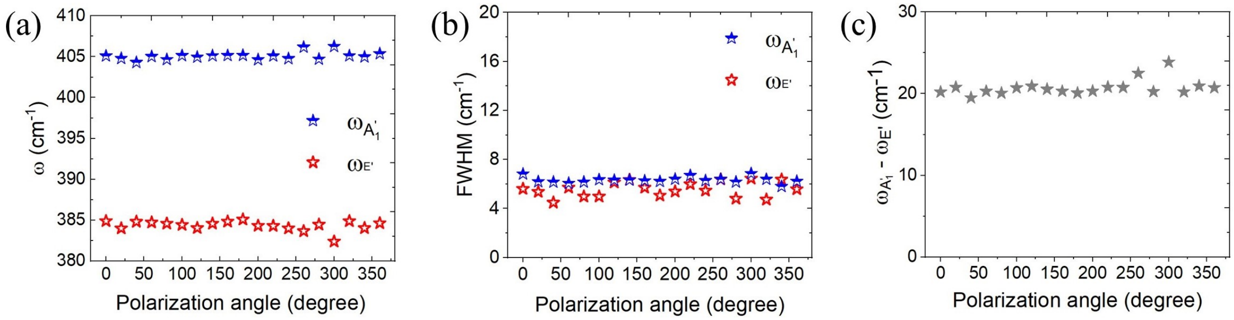

Figure 3: (a) Frequency for in-plane and out-of-plane modes, (b) their FWHM, and (c) $\omega_{A_1'}$ - $\omega_{E'}$ as a function of the incident polarization angle, respectively.

4. Intensities and linewidth maps from hyperspectral analysis

Figures 4 (a) and (b) illustrate the intensity maps for both Raman modes of MoS_2. As observed, the intensity of the E' mode varied less than 4 times in the regions inside and outside the slits. In contrast, we noted a 6-fold higher intensity difference in the same

regions for the A_1' mode, indicating that its intensity is more sensitive to variations across the sample.

Concerning the FWHM, figures 4 (c) and (d) show how this parameter changes in each region of the sample for the E' and A_1' modes, respectively, for both polarization conditions. For the E' mode, as can be seen in the figure 4 (c), we observed an increase in FWHM in the suspended regions, which is expected due to the changes in the lattice symmetry due to the applied uniaxial strain.[9] In the supported regions, the in-plane mode usually does not show an appreciable increase in the linewidth, since it is subjected to a biaxial strain.[1] One can see that this behavior occurs for both polarizations. In figure 4 (d), the FWHM maps for the A_1' mode are presented. In contrast to the previous case, the A_1' mode shows a larger linewidth in the supported regions, probably because in these regions the out-of-plane mode is influenced by both the strain and the doping mechanisms.[1,10] Similar to the E' case, the characteristic of FWHM for A_1' mode does not show a considerable dependence on the polarization.

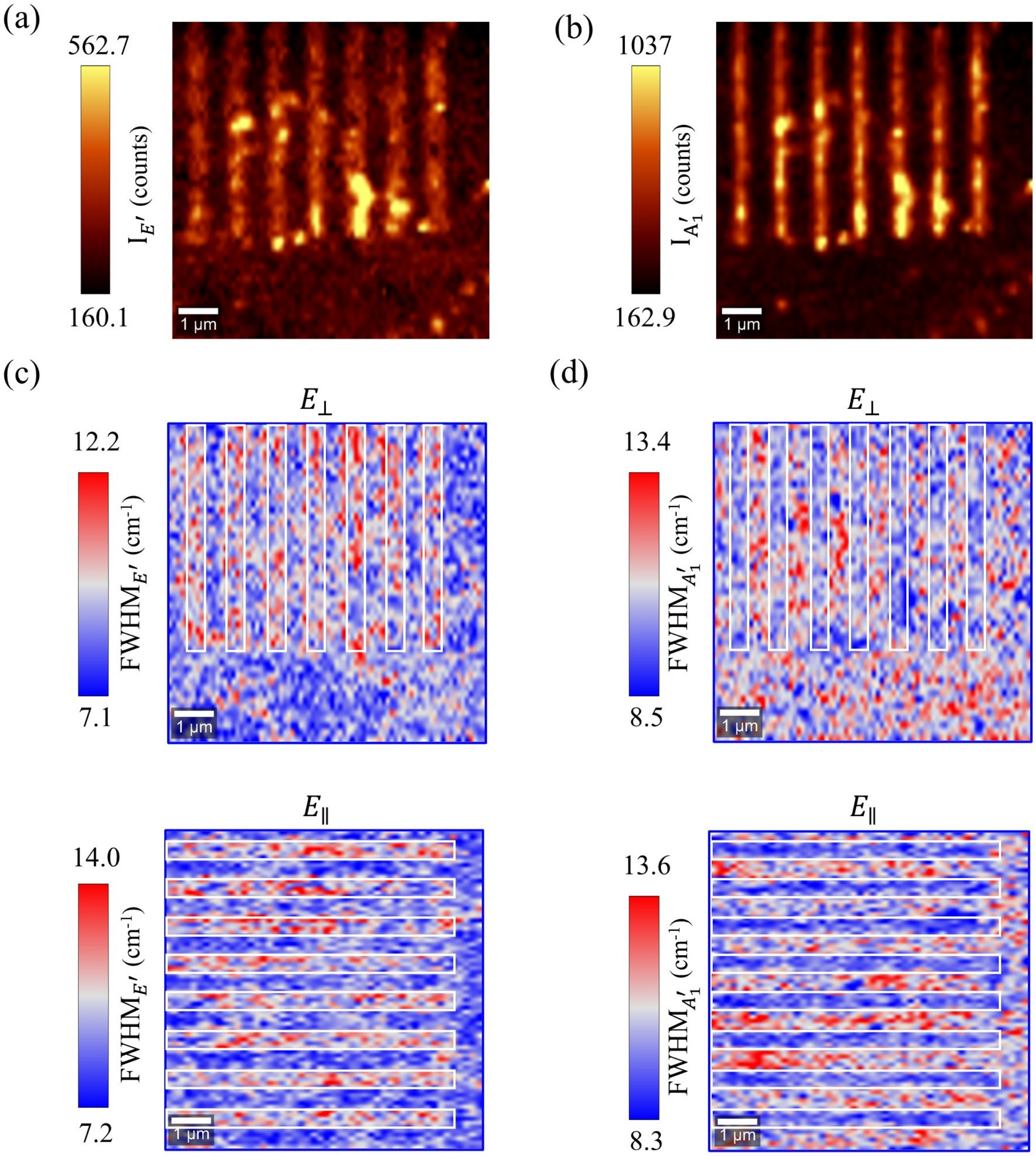

Figure 4: Intensity maps for (a) E' and (b) A_1'. Linewidth maps for (c) E' and (d) A_1', in both polarization conditions.

5. Numerical simulations regarding the grating protrusions

The protrusions near the edges of the slits occur due to Au redeposition during the etching of the metallic thin film. These unwanted geometries will deform the edges of the slits,

changing the regular corners to irregular ones. Simulating these irregular geometries to accurately replicate real corners is challenging. However, in practical terms, even with the edges presenting irregular shapes, they generate hot spots as well, meaning we still observe localized plasmons (LPs) in these regions. Figure 5 shows the numerical simulations for the electric field in the gold slits with regular corners (a) and protrusions (b). Additionally, the protrusion could deform the MoS_2 monolayer differently than in the regular corners, causing a strain that deviates from the ideal case. Nevertheless, mapping this strain gradient is very demanding. However, even though simulating the real physical case is difficult, our technique allows us to measure the average values of the strain and doping inside and outside the slit (including the irregular corners) region, distinguishing the values between them. These results are sufficient for us to observe the desired properties and indicate the plasmon-induced additional doping channel for a specific polarization of the incident radiation in the slits region.

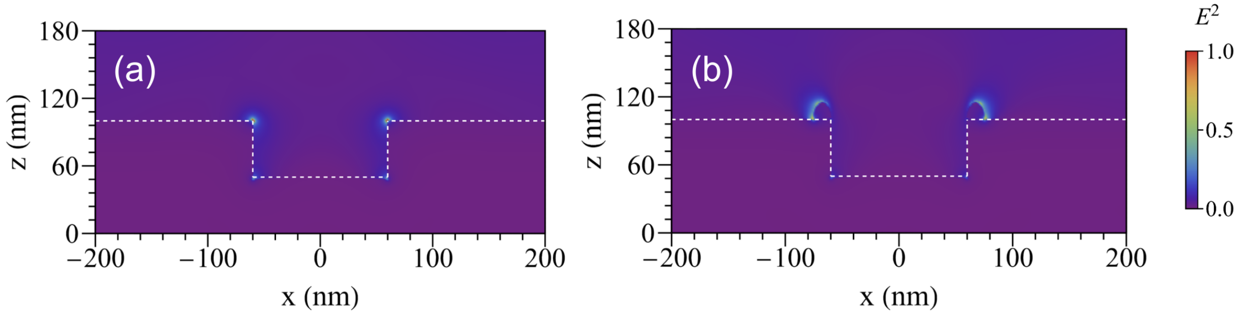

Figure 5: Numerical simulations for the electric field in the gold slits with regular corners (a) and protrusions (b).

References

(1) Velicky, M.; Rodriguez, A.; Bousa, M.; Krayev, A. V.; Vondracek, M.; Honolka, J.; Ahmadi, M.; Donnelly, G. E.; Huang, F.; Abruna, H. D.; others Strain and charge doping fingerprints of the strong interaction between monolayer MoS_2 and gold. *The Journal of Physical Chemistry Letters* **2020**, *11*, 6112–6118.

(2) Mandelbaum, Y.; Tkachev, M.; Sanjeev, A.; Zalevsky, Z.; Zitoun, D.; Karsenty, A. Tips versus Holes: $\times$ 10 Higher Scattering in FIB-made Plasmonic Nanoscale Arrays for Spectral Imaging. *ACS Omega* **2024**,

(3) Bertolazzi, S.; Brivio, J.; Kis, A. Stretching and breaking of ultrathin MoS_2. *ACS Nano* **2011**, *5*, 9703–9709.

(4) Li, T. Ideal strength and phonon instability in single-layer MoS_2. *Physical Review B* **2012**, *85*, 235407.

(5) Fair, K.; Ford, M. Phase transitions and optical properties of the semiconducting and metallic phases of single-layer MoS_2. *Nanotechnology* **2015**, *26*, 435705.

(6) Zhao, J.; Kou, L.; Jiang, J.-W.; Rabczuk, T. Tension-induced phase transition of single-layer molybdenum disulphide (MoS_2) at low temperatures. *Nanotechnology* **2014**, *25*, 295701.

(7) Chaudhuri, S.; Das, A. K.; Das, G. P.; Dev, B. N. Strain induced effects on the electronic and phononic properties of 2H and 1T′ monolayer MoS_2. *Physica B: Condensed Matter* **2023**, *655*, 414701.

(8) Yu, Y.; Nam, G.-H.; He, Q.; Wu, X.-J.; Zhang, K.; Yang, Z.; Chen, J.; Ma, Q.; Zhao, M.; Liu, Z.; others High phase-purity 1T′-MoS_2-and 1T′-$MoSe_2$-layered crystals. *Nature chemistry* **2018**, *10*, 638–643.

(9) Conley, H. J.; Wang, B.; Ziegler, J. I.; Haglund Jr, R. F.; Pantelides, S. T.; Bolotin, K. I. Bandgap engineering of strained monolayer and bilayer MoS_2. *Nano Letters* **2013**, *13*, 3626–3630.

(10) Panasci, S. E.; Schiliró, E.; Greco, G.; Cannas, M.; Gelardi, F. M.; Agnello, S.; Roccaforte, F.; Giannazzo, F. Strain, doping, and electronic transport of large area monolayer MoS_2 exfoliated on gold and transferred to an insulating substrate. *ACS Applied Materials & Interfaces* **2021**, *13*, 31248–31259.
